# LIM Zinc Finger Domain Containing 1 Risk Genotype of Recipient Is Associated with Renal Tubular Inflammation in Kidney Transplantation

**DOI:** 10.3390/genes15060773

**Published:** 2024-06-13

**Authors:** Yasar Caliskan, Yasemin Ozluk, Kento Kurashima, Safak Mirioglu, Ahmet Burak Dirim, Ozge Hurdogan, Ozgur Akin Oto, Marzena Syn, Mustafa Nazzal, Ajay Jain, John Edwards, Halil Yazici, Krista L. Lentine

**Affiliations:** 1 Division of Nephrology, SSM Saint Louis University Hospital, Saint Louis, MO 63110, USA; john.edwards@health.slu.edu (J.E.); krista.lentine@health.slu.edu (K.L.L.); 2 Division of Nephrology, Istanbul School of Medicine, Istanbul University, Istanbul 34093, Turkey; smirioglu@gmail.com (S.M.); ahmetburakdirim@gmail.com (A.B.D.); maviozgurluk@gmail.com (O.A.O.); halildrr@yahoo.com (H.Y.); 3 Department of Pathology, Istanbul School of Medicine, Istanbul University, Istanbul 34093, Turkey; yasozluk@gmail.com (Y.O.); ozgehurdogan@gmail.com (O.H.); 4 Department of Pediatrics, School of Medicine, SSM Saint Louis University, Saint Louis, MO 63104, USA; kento.kurashima@health.slu.edu (K.K.); mkzvet@gmail.com (M.S.); ajay.jain@slucare.ssmhealth.com (A.J.); 5 Department of Surgery, SSM Saint Louis University Hospital, Saint Louis, MO 63110, USA; mustafa.nazzal@health.slu.edu

**Keywords:** Banff classification, genetics, kidney transplantation, LIMS1, T-cell mediated rejection, tubulitis

## Abstract

Background: Homozygosity for *LIMS1* rs893403-GG genotype is linked to an increased risk of allograft rejection after kidney transplantation. Ischemia-reperfusion of the kidney allograft leads to long term infiltration of activated and effector-memory T lymphocytes and resulting in rejection and long-term fibrosis. However, the genotype, *LIMS1* expression under ischemic conditions and the long-term histopathological relationships remain ill-defined. Methods: We examined the impact of the recipient’s *LIMS1*-rs893403 genotype with transplant kidney histopathology. The association of the *LIMS1*-rs893403 genotype and *LIMS1* and *GCC2* mRNA expression in ischemic donor kidneys were also examined. Recipients who underwent transplant kidney biopsy were genotyped for the *LIMS1*-rs893403 variant and associated deletion. Histopathological findings were compared between recipients with *LIMS1* risk and non-risk genotypes. Real-time PCR and immunofluorescence staining for *LIMS1* and *GCC2* expression were performed in non-utilized donor kidneys. Results: Demographic, clinical, and treatment characteristics and the histopathological diagnosis were similar between recipients with rs893403 GG and AA/AG genotype. The Banff tubulitis score was higher in GG recipients (n = 24) compared to AA/AG (n = 86) recipients (1.42 ± 0.65 vs. 1.12 ± 0.66, *p* = 0.03). Ischemic kidneys with GG showed higher *LIMS1* and *GCC2* mRNA expression than kidneys with AG. Kidneys with rs893403-GG had higher tubular LIMS1 and GCC2 immunohistochemical staining compared to kidneys with rs893403-AG. Conclusions: Our data supports the role of the *LIMS1* locus in kidney transplant rejection, particularly in lymphocyte infiltration into the internal aspect of the tubular basement membranes. Increased *LIMS1* and *GCC2* expression in ischemic donor kidneys with the GG genotype require further studies.

## 1. Introduction

Human leukocyte antigen (HLA) matching between the donor and recipient has long been acknowledged as crucial for kidney transplant (KTx) survival but it does not fully explain the risk of rejection. Recent advances have highlighted significant roles for non-HLA antigen targets and mismatching in the risk of posttransplant rejection [1,2,3]. A genetic risk variant of the LIM zinc finger domain containing 1 (*LIMS*1) gene has been identified, with a suggestion of an increased rejection risk in KTx recipients with the homozygous risk allele rs893403-G tagging a deletion near *LIMS1* [2,4]. A robust linkage disequilibrium between rs893403-G, a tag single nucleotide variant (SNV), and the deletion CNVR915.1 as well as the cis-expression of grip and coiled-coil domain containing protein 2 (*GCC2*) gene was reported [2]. The expression quantitative trait loci (eQTL) kidney transcriptome data of NEPTUNE (Nephrotic Syndrome Study Network) study patients showed that rs893403-GG allele was associated with decreased *LIMS1* and *GCC2* expression in the tubulointerstitium and glomerulus, respectively [5]. The tag SNV, rs893403, resides in the intronic portion of *LIMS1* on chromosome 2q12.3. *GCC2* is a gene neighboring *LIMS1* that has a canonical role in late endosome-to-Golgi trafficking and stimulates peripheral blood lymphocyte transforming growth factor β (TGF-β) signaling by regulating mannose-6-phosphate trafficking [6,7,8]. Our group previously reported that kidney transplant recipients homozygous for the *LIMS1* rs893403-GG genotype had an approximately 2.5 times higher risk of T-cell-mediated rejection (TCMR) than those who did not have this genotype [9]. These results were compatible with the study by Steers et al. [2] showing a 63% higher risk of rejection for recipients with the homozygous *LIMS1* rs893403-GG genotype.

The Banff classification is the predominant classification system used for transplant biopsy diagnosis worldwide. It is necessary to characterize the histologic patterns of rejection linked to the *LIMS1* rs893403 risk genotype according to the Banff classification. Ischemia–reperfusion of the kidney allograft results in long-term infiltration of activated and effector–memory T lymphocytes, leading to rejection and chronic fibrosis. However, the *LIMS1* rs893403 genotype, *LIMS1* and *GCC2* expression under ischemic conditions and the long-term histopathological relationships remain ill-defined [10]. The effects of the rs893403 genotype on the expression of *LIMS1*, *GCC2* and associated genes in ischemic donor kidneys are also unclear. To date, data are lacking on detailed histopathological and gene expression associations of the *LIMS1* rejection risk allele. To address important knowledge gaps, we performed *LIMS1* rs893403 genotyping among a cohort of KTx recipients and examined relationships between the recipient’s *LIMS1* genotype, histopathological Banff classification of lesions and long-term outcomes. *LIMS1* and *GCC2* expression were also evaluated in ischemic human donor kidneys.

## 2. Materials and Methods

### 2.1. Study Population

The study included KTx recipients aged 18 years or older, followed at the Istanbul Faculty of Medicine Transplant Clinic and had DNA available for genotyping, underwent an allograft biopsy, and provided research consent. Allograft biopsies were performed “for cause” prompted by persistent, unexplained elevation in serum creatinine levels and/or proteinuria ≥ 1 g/day. In cases of repeat allograft biopsies from the same patient, the first allograft biopsy is evaluated. Exclusion criteria were having multiorgan transplantation, unable to provide consent or cognitive impairment. The DNA of recipients meeting the selection criteria underwent genotyping for the *LIMS1* rs893403 variant using Sanger sequencing, followed by confirmation of the deletion by PCR.

Data regarding patients’ demographic and clinical features [age, ethnicity, sex, primary kidney disease, kidney replacement therapy (KRT) modalities, duration of KRT, pretransplant sensitization history, panel-reactive antibody (PRA) levels, donor specific antibody (DSA), HLA mismatch, and transplant date] were extracted from medical records. De novo development of posttransplant DSAs at the time of biopsy was evaluated. Study patients received induction and maintenance immunosuppressive treatment according to the center’s protocol, as reported in previous studies [9,11]. Triple maintenance immunosuppressive regimen included a calcineurin inhibitor (CNI) (cyclosporine or tacrolimus), an antiproliferative drug [azathioprine (AZA) or mycophenolic acid (MPA) derivative (mycophenolate mofetil (MMF), sodium] and prednisolone (Pred). Target blood levels of cyclosporine (C0) and tacrolimus after transplantation were, respectively, 200–300 ng/mL and 8–12 ng/mL for the first three months, and 50–150 ng/mL and 4–8 ng/mL for subsequent months. MMF and AZA were administered at a dosage of 2 g/day (1440 mg/day for mycophenolate sodium) and 1.5 mg/kg/day, respectively. On postoperative day 1, patients received methylprednisolone beginning with a dose of 120 mg daily, with a rapid taper and reaching maintenance dose of 10 mg daily within the first month and 5 mg daily within the first year. Alterations were made in treatment strategies per immunologic risk and posttransplant complications, if necessary. Empiric anti-rejection treatment prior to biopsy was not given to any study patient.

The study procedures were carried out in accordance with good medical and laboratory practices and the principles outlined in the Declaration of Helsinki on biomedical research involving human subjects. The present study received approval from the Istanbul School of Medicine Clinical Studies Board (IRB approval number 2011/483-480). Written informed consent was obtained from all participants enrolled in the study.

### 2.2. Molecular Analyses

Peripheral blood samples were used for extraction of genomic DNA. *LIMS1* rs893403 genotyping was carried out via Sanger sequencing. The risk genotype was defined as homozygosity for the *LIMS1* rs893403-GG genotype in recipients. 

#### 2.2.1. PCR-Based Deletion Confirmation

The CNVR915.1 deletion was validated through quantitative PCR on genomic DNA from participants with rs893043-AG and GG genotypes. To confirm the deletion, the study utilized the primer pairs employed by Steers et al [2] with the presence of a PCR product between close primer pairs indicating the deletion. The specified primer pair produced a single band PCR product and Sanger sequencing of this amplicon, using the same primers, pinpointed the deletion breakpoints:

- CNV915.1-F: 5’-AAAGACCTCAAATCAATAGCCTG-3’
- CNVR915.1-R: 5’-GGACATTTAGGCTGCTTCTG-3’

#### 2.2.2. HLA Genotyping

PCR–sequence-specific oligonucleotide (PCRSSO) with Luminex technology was used to analyze HLA class I (HLA-A and HLA-B) and class II (HLA-DRB1) genotypes of patients and donors (One Lambda Inc., Canoga Park, CA, USA). The number of HLA mismatches between donors and recipients were calculated based on HLA A, B and DR loci.

#### 2.2.3. Anti-HLA Antibody Screening

Serum samples were analyzed for the presence of anti-HLA class I and II antibodies using Luminex kits (One Lambda Inc.). A positive reaction was defined by a normalized mean fluorescence intensity (MFI) value of 1000 or greater. For recipients with multiple measurements, the highest MFI value of donor specific anti-HLA class I and II antibodies after transplantation was included in the analysis.

#### 2.2.4. Biochemical Tests

Fasting serum samples for biochemical studies were obtained from all participants. Laboratory values, including complete blood cell count, serum levels of creatinine and albumin were measured using standard enzymatic procedures. The urinary protein-to-creatinine ratio (Up/c) from the first morning urine specimen was used to estimate quantitative proteinuria. 

### 2.3. Follow-Up Principles

Following a biopsy, patients were monitored at the kidney transplantation clinic, with follow-up intervals gradually extended to every three months. The follow-up period was defined as the time between allograft biopsy and patient’s last outpatient visit, transplant kidney failure or death, or the end of study (1 October 2020).

Patient charts were reviewed to retrieve laboratory data including complete blood count, serum albumin, creatinine, quantitative proteinuria and DSA levels. The estimated glomerular filtration rate (eGFR) was calculated using the 2009 equation of Chronic Kidney Disease Epidemiology Collaboration (CKD-EPI) [12]. Graft failure was defined as a return to dialysis, re-transplantation, or death with a functioning graft.

### 2.4. Histopathological Evaluation for Classifying Rejection

Kidney biopsy specimens were considered adequate if they contained seven or more glomeruli and at least two arteries. All histochemical and immunohistochemical staining were performed on 3–4 micrometer sections. For immunofluorescence staining (IgM, IgG, IgA, C3, C1q, lambda and kappa light chains, fibrinogen), a 0.4–0.6 cm unfixed tissue was frozen using liquid nitrogen. The remaining tissues was fixed in Hollande's fixative, embedded in paraffin, and routinely processed for light microscopic evaluation using periodic acid-Schiff, hematoxylin and eosin, Masson trichrome, methenamine silver-periodic acid and Congo red stains. Specimens were evaluated using the Banff criteria by light microscopy [13,14]. Immunohistochemistry was used to perform C4d staining on paraffin embedded tissue blocks, with positive results indicated by linear and circumferential staining in peritubular capillaries (C4d > 0) [13]. Transplant rejections were classified as either TCMR or antibody-mediated rejection (ABMR), according to Banff 2013 criteria [13]. The Banff scores for each lesion were compared between the GG and AA/AG genotypes. Tubulitis was scored from t1 to t3 according to the number of cells present per tubular cross-section in non-atrophic tubules [15].

### 2.5. RNA Extraction and Real-Time PCR Analysis

At Saint Louis University, non-utilized deceased donor kidneys (unsuitable for human KTx) underwent extraction of RNA, real time PCR analysis and immunohistochemical staining to understand the effects of ischemia on renal *LIMS*1 and *GCC2* expression. Non-utilized human kidneys with consent for research were procured from the tissue and organ recovery organization, Mid-America Transplant Center, under a material transfer agreement with Saint Louis University, following approval from Institutional Review Board and Institutional Biosafety Committee (Approval No. 2018-00040). Kidney biopsy samples were then obtained from four non-utilized deceased donor kidneys. RNA was extracted from these samples using an Allprep DNA/RNA mini kit (QIAGEN, Valencia, CA, USA). Then, reverse transcription was performed with a Verso cDNA Synthesis Kit (Thermo Fisher, Waltham, MA, USA). Real-time PCR analysis was conducted with a CFX Connect Real-Time Detection System (Bio-Rad, Hercules, CA, USA) and iTaq SYBR Green Supermix (Bio-Rad). The expression of *LIMS1* and *GCC2* was normalized to the housekeeping gene, *GAPDH*. Each assay was triplicated.

### 2.6. Immunohistochemistry

Ischemic kidney tissue antibody staining was performed with the use of mouse IgG1 to human LIMS1 (LSBio LS-C169391, Shirley, MA, USA) as well as rabbit polyclonal IgG antibody to human GCC2 (Genetex GTX51372, Alton Pkwy Irvine, CA, USA) on paraffin-embedded tissues with the use of heat-induced antigen retrieval.

### 2.7. Statistical Analyses

Data were presented as mean ± standard deviation (SD) for normally distributed or as median [interquartile range (IQR)] for non-normally distributed data. Nonparametric and parametric tests were employed based on the data distribution pattern. Continuous variables between the GG and AA/AG genotype groups were compared using the Mann–Whitney U test or the *t*-test, as appropriate. Proportional differences were evaluated by the Fisher’s exact test. Allograft survival according to AA/AG and GG genotype were analyzed via the Kaplan-Meier method, with allograft survival time computed from allograft biopsy to the last follow-up or the primary outcome. All statistical tests were two-sided with statistical significance set at *p* < 0.05. Statistical analyses were performed using SPSS software for Windows (SPSS version 25.0, IBM Corp., Armonk, NY, USA) and R version 3.5.2 [16].

## 3. Results

### 3.1. Study Population

There were 875 KTx recipients in the study period, of whom 127 had a biopsy available in the center’s database. Of these, 110 prevalent KTx recipients who underwent a kidney allograft biopsy met the study inclusion criteria (60.9% men; 79% living donor transplants; age: 30 ± 12 years). In this study, alleles A and G exhibited Hardy–Weinberg equilibrium with respective frequencies of *p* = 0.53 and q = 0.47. The *LIMS1* rs893403 GG, AG, and AA genotype distribution among KTx recipients revealed that GG, AG, and AA genotypes were observed in 21.8% (n = 24), 50.9% (n = 56), and 27.3% (n = 30) of participants, respectively. Recipients were categorized based on their *LIMS1* rs893403 variant genotype: the “risk genotype” group consisted of 24 recipients homozygous for the *LIMS1* rs893403-GG genotype, while the “non-risk genotype” group comprised 86 recipients with the *LIMS1* rs893403-AA or AG genotype. Furthermore, the CNVR915.1 deletion was confirmed through quantitative PCR in all participants’ samples with the rs893043-GG genotype (24/24). This confirmation was based on the presence of a PCR product between closely positioned primer pairs.

### 3.2. Clinical, Histopathologic, and Therapeutic Features

KTx recipients with GG and AA/AG genotypes were similar with regard to age, sex, pretransplant RRT, history of failed KTx, PRA level, donor sex, age and type, HLA mismatch number, induction and maintenance immunosuppressive treatments (Table 1). In all study patients, pretransplant DSA were negative. 

### 3.3. Allograft Biopsies

All selected patients underwent a for-cause allograft biopsy at a follow up time of median 6.2 years (IQR 2.5–6.2) after transplant. First, allograft biopsies of the patients were evaluated. The Banff 2013 scores obtained from transplant kidney biopsies were assessed and compared between recipients who were homozygous for the *LIMS1* rs893403-GG genotype (n = 24) and those with AA/AG genotypes (n = 86) (Table 2). The posttransplant time of biopsy was similar between the groups (*p* = 0.35). The mean serum creatinine levels, mean eGFR levels, and median proteinuria level at the time of allograft biopsy were not significantly different between the study groups (*p* = 0.73, *p* = 0.23, and *p* = 0.09, respectively). Overall, there was no difference regarding the number of patients with de novo DSAs between the study groups [GG group (n = 11, 45.8%) vs. AA/AG group (n = 30, 34.9%), *p* = 0.33].

A total of 90 (81.8%) biopsies showed rejection. Although not reaching statistical significance, the rates of TCMR and C4d-positive ABMR were higher in recipients with the GG genotype compared to the AA/AG genotypes [TCMR: n = 10 (42%) vs. n = 22 (26%), *p* = 0.13; C4d-positive ABMR, n = 11 (46%) vs. n = 24 (28%), *p* = 0.09] (Table 2). There were no differences in ABMR rates between patients with the GG and AA/AG genotypes (Table 2). Other diagnoses were recurrent/de novo glomerulonephritis (n = 6), CNI toxicity (n = 3), BK virus nephropathy (n = 1), diabetic nephropathy (n = 1) and amyloidosis (n = 1). There were also no differences in rates of these biopsy diagnosis between the study groups.

### 3.4. Banff Classification Scores

The mean Banff tubulitis score was significantly higher in the GG group compared to the AA/AG group (1.42 ± 0.65 vs. 1.12 ± 0.66, *p* = 0.03) (Table 2). There was no significant difference regarding the mean interstitial inflammation score between study groups (*p* = 0.57). Regarding ABMR lesions, mean glomerulitis, peritubular capillaritis, transplant glomerulopathy, C4d, interstitial fibrosis, and tubular atrophy scores were also similar between the GG and AA/AG groups (*p* = 0.41, *p* = 0.25, *p* = 0.77, *p* = 0.33, *p* = 0.58, and *p* = 0.45, respectively). The microvascular inflammation score (glomerulitis + peritubular capillaritis) was also similar between the study groups (*p* = 0.26).

### 3.5. LIMS1 and GCC2 Expression in Non-Utilized Ischemic Kidneys

**Figure 1:**
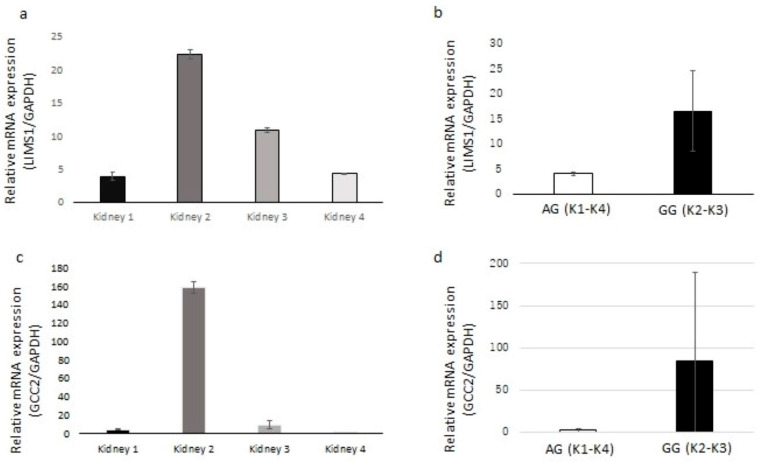
Relative mRNA expression of *LIMS1* and *GCC2* in RNA interference experiments by quantitative real-time PCR. (**a**) Relative mRNA expression ratio of LIMS1/the reference gene GAPDH in 4 ischemic donor kidneys. (**b**) The LIMS1/GAPDH mRNA expression ratio is higher in donor kidneys with the rs893403-GG genotype (K2–K3) compared with donor kidneys with the rs893403-AG genotype (K1–K4). (**c**) Relative mRNA expression ratio of GCC2/the reference gene GAPDH in 4 ischemic donor kidneys. (**d**) The LIMS1/GAPDH mRNA expression ratio is higher in donor kidneys with the rs893403-GG genotype (K2–K3) compared with donor kidneys with the rs893403-AG genotype (K1–K4). The cold ischemia time of donor kidneys are as follows: Kidney 1 (42 h), Kidney 2 (32 h), Kidney 3 (17 h), and Kidney 4 (39 h). Error bars represent the standard deviations (SDs).

*LIMS1* and *GCC2* gene expression in non-utilized ischemic deceased donor kidneys were examined by RT-PCR. The median cold ischemia time of the donor kidneys was 35.5 (IQR, 40.5–24.5) hours. The mRNA expression of *LIMS1* and *GCC2* in each kidney is shown in Figure 1. The expression of these two genes corresponded to each other. We compared *LIMS1* and *GCC2* gene expression between kidneys from donors with the AG and GG genotypes (Figure 1b,d). For both genes, kidneys with the GG genotype showed higher expression than kidneys with the AG genotype.

### 3.6. LIMS1 and GCC2 Immunohistochemical Staining of Non-Utilized Kidneys

**Figure 2:**
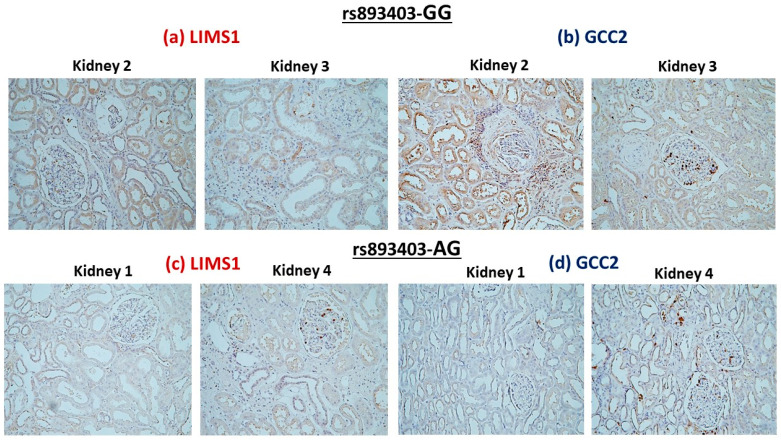
LIMS1 and GCC2 immunofluorescence staining in ischemic kidney sections from a donor with the *LIMS1* rs893403 GG genotype (**a**,**b**). LIMS1 and GCC2 immunofluorescence staining in ischemic kidney sections from a donor with the *LIMS1* rs893403 AG genotype (**c**,**d**).

Using immunohistochemical staining studies on non-utilized donor kidneys, we confirmed that LIMS1 and GCC2 were expressed in human kidneys. However, in deceased donor kidneys with homozygous rs893403-GG genotype (Figure 2a,b), LIMS1 and GCC2 staining were significant in tubulointerstitial area compared to deceased donor kidneys with rs893403-AG genotype (Figure 2c,d). 

### 3.7. Follow-Up and Outcomes

A total of 56 (50.9%) recipients lost their allografts after a median post-biopsy follow up time of 3.2 years (IQR 0.8–6.9). Kaplan–Meier estimates of 5-year (83.3% vs. 80.2%, *p* = 0.55) and 10-year graft survival rates (62.5% vs. 67.4%, *p* = 0.98) did not differ significantly in those with the GG genotype compared to the AA/AG groups. Kaplan–Meier estimates of 5-year (GG, 83.3% vs. AA/AG 81.2%, *p* = 0.63) and 10-year death-censored graft survival rates (GG, 62.5% vs. AA/AG 70.6%, *p* = 0.75) also did not differ significantly by genotype.

## 4. Discussion

In our study of 110 transplant recipients with for-cause transplant kidney biopsy and *LIMS1* rs893403 genotyping, those with the homozygous *LIMS1* rs893403-GG genotype had significantly higher tubulitis scores. Previous kidney transcriptome data showed that rs893403-GG was associated with decreased expression of *LIMS1* in tubules and *GCC2* in the glomerulus in non-ischemic kidneys. However, in the present study ischemic kidneys with -GG genotype showed higher *LIMS1* and *GCC2* expression and protein staining than kidneys with AG genotype. Although the TCMR rates did not reach to statistically significant difference between recipient *LIMS1* rs893403 genotypes, the novel observation of higher tubulitis scores in recipients with *LIMS1* GG genotype and donor gene expression profiles support the role of the locus including *LIMS1* and *GCC2* genes in kidney allograft rejection.

The identification of mononuclear cells in the basolateral aspect of the renal tubule epithelium is a primary diagnostic criterion used within the Banff schema for acute TCMR [17]. Previous studies suggested several SNVs linked to acute rejection based on the tubulitis severity on transplant kidney biopsies [18]. The protein encoded by *PRDM1* SNV rs811925 known to repress beta-interferon gene expression. Interestingly, the presence of the rs811925 C allele was associated with increased likelihood of a tubulitis score > 2 compared to a t-score <1 [19]. Additionally, in the same study, a missense SNV (rs2228059) in *IL15RA*, a receptor for the inflammatory cytokine interleukin 15, was found to be associated with tubulitis severity [19]. However, there was a significant center-to-center variation in the diagnosis of acute rejection in this study that can explain the difficulty in validating SNVs or other genomics markers in different centers.

Recently, in a large transplant cohort, Steers et al. [2] reported that recipient-donor *LIMS1* locus genomic mismatch was associated with an increased allograft rejection risk [2]. Consistent with this report, our group also found that the *LIMS1* risk genotype rs893403-GG in KTx recipients was significantly associated with TCMR compared to the non-risk *LIMS1* genotype, while ABMR and allograft failure rates were similar in both groups [2]. Previous eQTL data showed that rs893403 has a direction-consistent cis-eQTL effect on the tubulointerstitial *LIMS1* messenger RNA (mRNA) level, and an association with regulatory T-cell *GCC2* expression and TGF-β-SMAD signaling was also reported [20,21]. The DICE (Database of Immune Cell Expression, Expression Quantitative Trait Loci and Epigenomics) project data showed that rs893043 may have a profound impact on naive CD4 regulatory T-cell *GCC2* expression, with lower expression for the GG allele compared to the AA and AG alleles [22]. The GTEx (Genotype–Tissue Expression) data also showed various tissue expression profiles that were mainly associated with the *GCC2* gene. In the current study, we observed that *LIMS1* and *GCC2* gene expression corresponded to each other in ischemic kidneys with both the GG and AG genotypes. Interestingly, in ischemic kidneys with the GG genotype, *LIMS1* and *GCC2* expression were higher than in kidneys with the AG genotype. Ischemia may cause different expression patterns of *LIMS1* and *GCC2* compared to previous databases.

In the present study, the association between the *LIMS1* rs893403-GG risk genotype and Banff tubulitis score severity is a novel observation. Tubulitis is an interaction of donor tubule epithelial cells and the recipient’s inflammatory cells. This is the first study evaluating the mRNA expression of *LIMS1* and *GCC2* in ischemic donor kidneys. *LIMS1* encodes a protein that is involved in cell adhesion and integrin signaling and is found in focal adhesion plaques. Data from the NephVS eQTL Browser (NephQTL) based on published kidney transcriptomes indicated that the rs893403-GG was associated with reduced expression of *LIMS1* in tubules and *GCC2* in the glomerulus [5]. Interestingly, analysis of the eQTL function in immune cell transcriptomes from published data revealed that rs893403 was not an eQTL for *LIMS1* in peripheral blood mononuclear cells (PBMCs) [22,23]. Instead, it demonstrated significant eQTL function on *GCC2*, the gene neighboring *LIMS1* at the 5’-end. Two mechanistic models may explain tubular inflammation during TCMR. The first one may be that a donor–recipient genotype mismatch triggers increased *LIMS1* and *GCC2* expression in allograft tubules and results in inflammation. In our study, we could not evaluate the donor–recipient genotype mismatch and expression profile. However, the increased expression pattern of *LIMS1* and *GCC2* in ischemic GG kidneys compared to ischemic AG kidneys require further studies. In the alternative mechanism, recipient regulatory T-cell *GCC2* expression might have a role in rejection-related tubule inflammation and tubulitis [24]. 

Although the rate of T-cell rejection was numerically higher in kidneys with the GG phenotype, these differences were not statistically significant. This could be due to the small sample size or it might be coincidental, as a previous study could not validate the effect of the *LIMS1* variant on allograft rejection. However, the observation of high tubulitis scores in recipients with the GG phenotype is an important finding that supports the roles of *LIMS1* and *GCC2* in tubular epithelial cells [25]. A limitation of our study is the lack of LIMS1 and GCC2 antibody staining in allograft biopsies with active TCMR. To better understand the alloimmune response associated with the *LIMS1* rs893403 locus, it is important to examine *LIMS1* and *GCC2* expression in peripheral blood and the transplanted kidney during active rejection. The lack of donor genotype data limits us from examining the effects of genomic mismatch on allograft histopathology.

In summary, this study provides additional evidence for the role of the *LIMS1* genotype in an important signature of T-cell-mediated kidney allograft rejection, tubulitis. Although TCMR rates were not statistically different between *LIMS1* genotypes, recipients with the homozygous *LIMS1* rs893403-GG genotype had higher tubulitis scores. Ischemia results different transcriptomic response in kidneys with different rs893403 genotype. A better understanding of the interplay of the *LIMS1* risk genotype, peripheral blood and kidney *LIMS1* and *GCC2* expression, and KTx allograft tubulitis requires further studies.

## Figures and Tables

**Table 1:** Demographic, clinical, and therapeutic features of the study groups based on the *LIMS1* gene rs893403 genotype.

	GG Genotype (n = 24)	AA/AG Genotype (n = 86)	*p* Value
Age (years), mean ± SD	30 ± 12	32 ± 12	0.41
Sex (M/F), n (%)	18 (75%)/6 (25%)	49 (57%)/37 (43%)	0.11
Pretransplant dialysis, n (%)			
Preemptive	2 (8.3%)	12 (14%)	
HD	17 (70.8%)	61 (70.9%)	
PD	4 (16.7%)	6 (7%)	0.42
HD + PD	1 (4.2%)	7 (8.1%)	
Previous kidney transplant, n (%)	1 (4.2%)	2 (2.3%)	0.62
Pretransplant last PRA level, n (%)			
<10%	24 (100%)	83 (96.5%)	
10% to 79%	-	3 (3.5%)	0.35
≥80%	-	-	
Donor age (years), mean ± SD	43 ± 12	42 ± 13	0.83
Donor sex (M/F), n (%)	16 (67%)/8 (33%)	47 (55%)/39 (45%)	0.29
Donor type (living/deceased), n (%)	17 (71%)/7 (29%)	70 (81%)/16 (19%)	0.26
HLA mismatches, mean ± SD	3.4 ± 1.1	3.0 ± 1.2	0.07
*Induction treatment*, *n* (%)			
ATG	7 (29%)	4 (16%)	0.51
IL2rAb	4 (17%)	16 (19%)	
No induction	13 (54%)	56 (65%)	
*Maintenance immunosuppression*, *n* (%)			
Tac + MMF/AZA + Pred	8 (33.3%)	28 (32.6%)	
CsA + MMF/AZA + Pred	12 (50%)	39 (45.3%)	
mTORi-based	2 (8.3%)	8 (9.3%)	0.97
AZA/MMF + Pred	2 (8.3%)	8 (9.3%)	
CNI + MMF/AZA	-	2 (2.3%)	
CNI + Pred	-	1 (1.2%)	

*Abbreviations*: ATG—antithymocyte globulin (rabbit), AZA—azathioprine, CNI—calcineurin inhibitor, CsA—cyclosporine, F—female, HD—hemodialysis, HLA—human leukocyte antigen, IL2rAb—interleukin-2 (IL-2) receptor-blocking antibodies, IQR—interquartile range, M—male, MMF—mycophenolate mofetil/sodium, mTORi—mammalian target of rapamycin inhibitor, PD—peritoneal dialysis, PRA—panel-reactive antibody, Pred—prednisone, SD—standard deviation, Tac—tacrolimus.

**Table 2:** Laboratory and histopathological characteristics of the study groups.

	GG Genotype (n = 24)	AA/AG Genotype (n = 86)	*p* Value
Allograft biopsy time after KTx (years), median (IQR)	7.6 (4.0–12.8)	5.4 (1.9–12.8)	0.35
Serum creatinine (mg/dL), median (IQR)	2.2 (1.5–2.5)	2.2 (1.68–2.8)	0.84
Proteinuria (g/day), median (IQR)	0.25 (0.03–1.43)	1 (0–2.5)	0.33
DSA, n (%)	11 (45.8%)	30 (34.9%)	0.33
Baseline biopsy results			
*Morphologic TCMR lesions and scores*			
Tubulitis score ≥ 1, n (%)	24 (100%)	77 (89.5%)	0.09
Tubulitis score, mean ± SD	1.42 ± 0.65	1.12 ± 0.66	**0.03**
Interstitial inflammation ≥ 1, n (%)	21 (87.5%)	70 (91.9%)	0.51
Interstitial inflammation, mean ± SD	1.33 ± 0.76	1.27 ± 0.68	0.57
*Morphologic ABMR lesions and scores*			
Glomerulitis score ≥ 1, n (%)	14 (58.3%)	41 (47.7%)	0.36
Glomerulitis score, mean ± SD	0.96 ± 1.04	0.78 ± 0.96	0.41
Peritubular capillaritis score ≥ 1, n (%)	17 (70.8%)	50 (58.1%)	0.26
Peritubular capillaritis score, mean ± SD	1.21 ± 1.06	0.93 ± 0.96	0.25
Microvascular inflammation (glomerulitis + peritubular capillaritis) score, mean ± SD	2.17 ± 1.86	1.71 ± 1.70	0.26
Transplant glomerulopathy score ≥ 1, n (%)	9 (37.5%)	33 (38.4%)	0.94
Transplant glomerulopathy score, mean ± SD	0.54 ± 0.83	0.64 ± 0.93	0.77
C4d in peritubular capillaritis (≥1), n (%)	12 (50%)	29 (34%)	0.15
C4d score, mean ± SD	1.04 ± 1.23	0.86 ± 1.28	0.33
Interstitial fibrosis score ≥ 1, n (%)	22 (91.7%)	74 (86%)	0.58
Interstitial fibrosis score, mean ± SD	1.25 ± 0.61	1.16 ± 0.65	0.58
Tubular atrophy score ≥ 1, n (%)	23 (94%)	78 (91%)	0.42
Tubular atrophy score, mean ± SD	1.21 ± 0.51	1.29 ± 0.63	0.45
Banff 2013 rejection types and categories			
Acute/active TCMR, n (%)	10 (42%)	22 (26%)	0.13
Acute/active ABMR, n (%)	10 (42%)	33 (38%)	0.77
Chronic/active ABMR, n (%)	4 (17%)	11 (14%)	0.79
C4d-positive ABMR, n (%)	11 (46%)	24 (28%)	0.09
Banff borderline lesion, n (%)	3 (13%)	16 (19%)	0.48
TCMR + ABMR, n (%)	4 (17%)	7 (8%)	0.22
Recurrent/de novo GN, n (%)	1 (4%)	5 (6%)	0.75
CNI toxicity, n (%)	-	3 (4%)	0.35
BKV nephropathy, n (%)	-	1 (1%)	0.60
Diabetic nephropathy, n (%)	-	1 (1%)	0.60
Amyloidosis, n (%)	-	1 (1%)	0.60

*Abbreviations:* ABMR—antibody-mediated rejection, BKV—BK virus, CNI toxicity—calcineurin inhibitor toxicity, IQR—interquartile range, KTx—kidney transplant, DSAs—donor-specific antibodies, TCMR—T-cell-mediated rejection.

## Data Availability

The original contributions presented in the study are included in the article, further inquiries can be directed to the corresponding author.

## Abbreviations

ABMR: antibody-mediated rejection, ATG: antithymocyte globulin (rabbit), AZA: azathioprine, CKD-EPI: Chronic Kidney Disease Epidemiology Collaboration, CNI: calcineurin inhibitor, CsA: cyclosporine, DICE: Database of Immune Cell Expression, Expression Quantitative Trait Loci and Epigenomics, DSA: donor-specific antibody, eGFR: estimated glomerular filtration rate, eQTL: expression quantitative trait locus, F: female, GCC2: grip and coiled-coil domain containing protein 2, GTEx: Genotype–Tissue Expression, HD: hemodialysis, HLA: human leukocyte antigen, IL2rAb: interleukin-2 (IL-2) receptor-blocking antibody, IQR: interquartile range, KTx: kidney transplantation, KRT: kidney replacement treatment, LIMS: LIM zinc finger domain containing 1, M: male, MMF: mycophenolate mofetil/sodium, mRNA: messenger RNA, mTORi: mammalian target of rapamycin inhibitor, NephQTL: NephVS eQTL Browser, PBMC: peripheral blood mononuclear cell, PD: peritoneal dialysis, PRA: panel-reactive antibody, Pred: prednisone, SD: standard deviation, SNP: single nucleotide polymorphism, SNV: single nucleotide variant, Tac: tacrolimus, TCMR: T-cell-mediated rejection, TGF-β: transforming growth factor β.

## References

[1] Li L., Sigdel T., Vitalone M., Lee S.H., Sarwal M. (2010). Differential immunogenicity and clinical relevance of kidney compartment specific antigens after renal transplantation. J. Proteome Res..

[2] Steers N.J., Li Y., Drace Z., D’Addario J.A., Fischman C., Liu L., Xu K., Na Y.J., Neugut Y.D., Zhang J.Y. (2019). Genomic Mismatch at LIMS1 Locus and Kidney Allograft Rejection. N. Engl. J. Med..

[3] Thomas C.P., Daloul R., Lentine K.L., Gohh R., Anand P.M., Rasouly H.M., Sharfuddin A.A., Schlondorff J.S., Rodig N.M., Freese M.E. (2023). Genetic evaluation of living kidney donor candidates: A review and recommendations for best practices. Am. J. Transplant..

[4] Zanoni F., Kiryluk K. (2020). Genetic background and transplantation outcomes: Insights from genome-wide association studies. Curr. Opin. Organ Transplant..

[5] Gillies C.E., Putler R., Menon R., Otto E., Yasutake K., Nair V., Hoover P., Lieb D., Li S., Eddy S. (2018). An eQTL Landscape of Kidney Tissue in Human Nephrotic Syndrome. Am. J. Hum. Genet..

[6] Reddy J.V., Burguete A.S., Sridevi K., Ganley I.G., Nottingham R.M., Pfeffer S.R. (2006). A functional role for the GCC185 golgin in mannose 6-phosphate receptor recycling. Mol. Biol. Cell.

[7] Brown F.C., Schindelhaim C.H., Pfeffer S.R. (2011). GCC185 plays independent roles in Golgi structure maintenance and AP-1-mediated vesicle tethering. J. Cell Biol..

[8] Burguete A.S., Fenn T.D., Brunger A.T., Pfeffer S.R. (2008). Rab and Arl GTPase family members cooperate in the localization of the golgin GCC185. Cell.

[9] Caliskan Y., Karahan G., Akgul S.U., Mirioglu S., Ozluk Y., Yazici H., Demir E., Dirim A.B., Turkmen A., Edwards J. (2021). LIMS1 risk genotype and T cell-mediated rejection in kidney transplant recipients. Nephrol. Dial. Transplant..

[10] Ascon M., Ascon D.B., Liu M., Cheadle C., Sarkar C., Racusen L., Hassoun H.T., Rabb H. (2009). Renal ischemia-reperfusion leads to long term infiltration of activated and effector-memory T lymphocytes. Kidney Int..

[11] Sarihan I., Caliskan Y., Mirioglu S., Ozluk Y., Senates B., Seyahi N., Basturk T., Yildiz A., Kilicaslan I., Sever M.S. (2020). Amyloid A Amyloidosis After Renal Transplantation: An Important Cause of Mortality. Transplantation.

[12] Levey A.S., Stevens L.A., Schmid C.H., Zhang Y.L., Castro A.F., Feldman H.I., Kusek J.W., Eggers P., Van Lente F., Greene T. (2009). A new equation to estimate glomerular filtration rate. Ann. Intern. Med..

[13] Haas M., Sis B., Racusen L.C., Solez K., Glotz D., Colvin R.B., Castro M.C., David D.S., David-Neto E., Bagnasco S.M. (2014). Banff 2013 meeting report: Inclusion of c4d-negative antibody-mediated rejection and antibody-associated arterial lesions. Am. J. Transplant..

[14] Racusen L.C., Colvin R.B., Solez K., Mihatsch M.J., Halloran P.F., Campbell P.M., Cecka M.J., Cosyns J.P., Demetris A.J., Fishbein M.C. (2003). Antibody-mediated rejection criteria—An addition to the Banff 97 classification of renal allograft rejection. Am. J. Transplant..

[15] Lusco M.A., Fogo A.B., Najafian B., Alpers C.E. (2016). AJKD Atlas of Renal Pathology: Acute T-Cell-Mediated Rejection. Am. J. Kidney Dis..

[16] R Core Team (2013). R: A Language and Environment for Statistical Computing.

[17] Racusen L.C., Halloran P.F., Solez K. (2004). Banff 2003 meeting report: New diagnostic insights and standards. Am. J. Transplant..

[18] Israni A., Leduc R., Holmes J., Jacobson P.A., Lamba V., Guan W., Schladt D., Chen J., Matas A.J., Oetting W.S. (2010). Single-nucleotide polymorphisms, acute rejection, and severity of tubulitis in kidney transplantation, accounting for center-to-center variation. Transplantation.

[19] Barnes N.A., Stephenson S.J., Tooze R.M., Doody G.M. (2009). Amino acid deprivation links BLIMP-1 to the immunomodulatory enzyme indoleamine 2,3-dioxygenase. J. Immunol..

[20] Wong M.G., Panchapakesan U., Qi W., Silva D.G., Chen X.M., Pollock C.A. (2011). Cation-independent mannose 6-phosphate receptor inhibitor (PXS25) inhibits fibrosis in human proximal tubular cells by inhibiting conversion of latent to active TGF-beta1. Am. J. Physiol. Renal. Physiol..

[21] Hassan A.B. (2003). Keys to the hidden treasures of the mannose 6-phosphate/insulin-like growth factor 2 receptor. Am. J. Pathol..

[22] Schmiedel B.J., Singh D., Madrigal A., Valdovino-Gonzalez A.G., White B.M., Zapardiel-Gonzalo J., Ha B., Altay G., Greenbaum J.A., McVicker G. (2018). Impact of Genetic Polymorphisms on Human Immune Cell Gene Expression. Cell.

[23] Consortium G.T. (2013). The Genotype-Tissue Expression (GTEx) project. Nat. Genet..

[24] Mirioglu S., Kiran B., Lentine K.L., Edwards J.C., Caliskan Y. (2024). Regulatory T cells in kidney transplant recipients with LIMS1 rs893403 risk genotype. Clin. Transplant..

[25] Markkinen S., Helantera I., Lauronen J., Lempinen M., Partanen J., Hyvarinen K. (2022). Mismatches in Gene Deletions and Kidney-related Proteins as Candidates for Histocompatibility Factors in Kidney Transplantation. Kidney Int. Rep..

